# Association of insulin resistance and fracture risk in the Osteoporotic Fractures in Men (MrOS) cohort

**DOI:** 10.1210/jendso/bvag101

**Published:** 2026-05-04

**Authors:** Jeonghoon Ha, Li-Yung Lui, Kristine Ensrud, Qian Xiao, Andrew R Hoffman, Joy Y Wu

**Affiliations:** Division of Endocrinology, Stanford University School of Medicine, Stanford, CA 94305, USA; Division of Endocrinology and Metabolism, Department of Internal Medicine, Seoul St. Mary's Hospital, College of Medicine, The Catholic University of Korea, Seoul, Korea; San Francisco Coordinating Center, California Pacific Medical Center, San Francisco, CA 94159, USA; Epidemiology & Community Health and Medicine, University of Minnesota, Minneapolis, MN 55455, USA; Department of Epidemiology, School of Public Health, University of Texas Health Science Center, Houston, TX 77030, USA; Division of Endocrinology, Stanford University School of Medicine, Stanford, CA 94305, USA; Division of Endocrinology, Stanford University School of Medicine, Stanford, CA 94305, USA

**Keywords:** insulin resistance, bone fractures, men's health, aged, cohort study

## Abstract

**Context:**

Insulin resistance (IR) is more prevalent in men than in women, but evidence directly linking IR to fracture risk in older men remains limited.

**Objective:**

To evaluate the association between insulin resistance (IR), estimated using the Homeostatic Model Assessment of Insulin Resistance (HOMA-IR) and the triglyceride–glucose (TyG) index, and fracture risk in older men.

**Methods:**

Data from 5994 men (≥65 years) in the MrOS cohort were analyzed. A total of 5416 participants were included in the HOMA-IR analysis and 5384 in the TyG index analysis. Fractures were self-reported and confirmed radiographically. Participants were grouped into quartiles based on IR indices, and fracture outcomes were evaluated during a mean follow-up of 12.2-13.6 years using Cox models.

**Results:**

A U-shaped association was observed in the HOMA-IR analysis, with Q3 vs Q1 showing a lower hazard ratio (HR) for any fracture (HR 0.82, 95% CI 0.70-0.97). Similarly, in the TyG index analysis, Q3 vs Q1 was associated with lower risks of any fracture (HR 0.84, 95% CI 0.72-0.98) and major osteoporotic fracture (MOF) (HR 0.80, 95% CI 0.65-0.99). No associations were found for hip or vertebral fractures, and results were unchanged in sensitivity analyses excluding T2DM.

**Conclusion:**

Older community-dwelling men in Q3 of HOMA-IR and TyG had modestly lower risks of any clinical fracture and MOF, but associations were inconsistent across quartiles and fracture types. Further studies in other populations are needed to confirm these findings.

Insulin resistance (IR) is a key pathophysiological feature of metabolic disorders that contributes to the development of type 2 diabetes mellitus (T2DM), cardiovascular disease, metabolic dysfunction-associated fatty liver disease, and other metabolic disorders [1-3]. As the prevalence of these disorders continues to increase, accessible tools for assessing IR are becoming increasingly relevant, particularly in research settings, while their clinical applicability requires further validation. The Homeostatic Model Assessment of Insulin Resistance (HOMA-IR) and the triglyceride–glucose (TyG) index, derived from fasting glucose, insulin, and triglyceride levels, are widely recognized surrogate markers of IR [4-6]. These indices are reliable biomarkers of IR and are commonly used in epidemiological research and clinical risk stratification [7].

In addition to its metabolic implications, several studies have examined the association between IR and bone health outcomes [8-11]. However, studies investigating the effects of IR on bone mineral density (BMD) and fracture risk have reported inconsistent results. Fractures, particularly in older adults, pose a significant public health challenge owing to their association with increased morbidity, mortality, and healthcare costs. However, evidence directly linking IR to fracture risk remains limited, especially in older populations, underscoring the need for further investigation. Considering that IR is more prevalent in men than in women, understanding its effect on skeletal health in older men is particularly important.

Most studies examining the relationship between IR and fractures have focused on mixed-sex or female-specific populations, such as postmenopausal women. Although these studies have enhanced our understanding of the association between IR and bone health, they have not fully addressed the effect of IR on fractures in men. Differences in fracture patterns and metabolic profiles between men and women suggest that findings from female-dominated or mixed-sex cohorts may not be directly applicable to men.

To address these gaps, the primary aim of this study was to investigate the association between IR, as assessed using HOMA-IR and the TyG index, and fracture risk in a well-defined population-based cohort of community-dwelling older men with extended follow-up.

## Materials and methods

### Study population

The Osteoporotic Fractures in Men (MrOS) Study is a multicenter, prospective, observational cohort study designed to evaluate the risk factors for fractures and osteoporosis in older men. In total, 5994 community-dwelling older men were recruited between March 2000 and April 2002, from 6 clinical sites across the United States: Birmingham, Alabama; Minneapolis, Minnesota; Palo Alto, California; Monongahela Valley, Pennsylvania; Portland, Oregon; and San Diego, California, as previously described [12, 13]. Eligible participants were men aged ≥65 years, able to walk unassisted, and without a history of bilateral hip replacement surgery [12, 13]. For this analysis, 5416 participants with baseline data available for HOMA-IR calculation and 5384 participants with data available for TyG index calculation were included. The study was approved by the institutional review boards of all participating institutions, and written informed consent was obtained from all participants prior to data collection. Further details regarding the MrOS study are available at https://mrosonline.ucsf.edu [12].

### Assessment of HOMA-IR and TyG index

Blood samples were collected at the baseline visit after an overnight fast. Serum samples were processed within 1 hour of collection, frozen, and stored at −80 °C until analysis. Glucose was measured using the hexokinase method, and insulin was analyzed with a 2-site immunoenzymometric assay utilizing autoanalyzers (Northwest Lipid Metabolism and Diabetes Research Laboratories, Seattle, WA). Triglycerides were measured using a Roche COBAS Integra 800 automated analyzer (Roche Diagnostics Corp., Indianapolis, IN) at the Oregon Veterans Affairs Clinical Laboratory. The HOMA-IR and TyG index, respectively, were calculated using the following formulae [14, 15]:

-
$\mathrm{HOMA}-\mathrm{IR}=\frac{\mathrm{Fasting}\;\mathrm{Insulin}\;(\mu U/\mathrm{mL})\;X\;\mathrm{Fasting}\;\mathrm{Glucose}\;(\mathrm{mg}/\mathrm{dL})\;}{405}$

-
$\mathrm{TyG}\;\mathrm{index}=\frac{\ln\;(\mathrm{Fasting}\;\mathrm{Triglycerides}\;(\mathrm{mg}/\mathrm{dL})\;X\;\mathrm{Fasting}\;\mathrm{Glucose}\;(\mathrm{mg}/\mathrm{dL}))}{2}$

HOMA-IR is the most widely used surrogate marker of IR [16, 17]. The TyG index is a simple, readily available, and reliable marker of IR [15, 18] and is associated with increased risks of mortality, cardiovascular diseases, carotid atherosclerosis, coronary artery disease, metabolic syndrome, and T2DM [7, 19-21]. Its diagnostic accuracy has been validated in multiple studies, showing a sensitivity of up to 96% using the hyperinsulinemic-euglycemic clamp and a specificity of up to 99% compared with HOMA-IR [22]. Higher values of both HOMA-IR and the TyG index indicate greater degrees of IR [7].

### Assessment of fractures

Incident fractures were self-reported every 4 months via mailed surveys and verified by radiographic reports, as described previously [23, 24]. Fractures due to malignancy were excluded from the analyses. The follow-up period was defined as the time from the baseline visit to the occurrence of a fracture, death, or the end of the study on August 1, 2024, whichever occurred first. Follow-up times were calculated for each fracture outcome separately, including any fracture, hip fracture, clinical vertebral fracture, and major osteoporotic fracture (MOF). Fracture outcomes were defined as follows: “any fracture” included all clinical fractures occurring during the follow-up period, and MOF comprised clinical fractures of the vertebrae, hip, wrist, or shoulder. The mean follow-up times were 12.2 years for any fracture, 13.6 years for hip and vertebral fractures, and 13.1 years for MOF.

### Assessment of covariates

BMD was measured at baseline using dual-energy X-ray absorptiometry (DXA) on Hologic QDR 4500-W densitometers (Hologic, Waltham, MA, United States). To ensure consistency across the 6 clinical sites, standardized scanning protocols were implemented, DXA operators were certified, and quality control of scan acquisition and analysis was monitored centrally. A set of phantoms was measured on all machines prior to baseline, and each scanner was monitored for longitudinal changes using regular measurements of spine and hip phantoms. Corrections to the BMD data were not required. The intraclinic coefficients of variation (CVs) ranged from 0.34% to 0.42% for spine phantoms and from 0.37% to 0.58% for hip phantoms. The interclinic CVs were 0.6% for spine and 0.9% for hip, with maximum differences in mean values of 1.4% (spine) and 2.2% (hip). Body weight and height were measured using standard equipment, and the body mass index (BMI) was calculated as weight (kg) divided by height (m^2^). At baseline, information regarding demographic, anthropometric, personal, and family medical history; lifestyle; and functional status was obtained through self-report, interview, or examination by trained and certified staff [12]. Prevalent fracture was defined as any fracture occurring after the age of 50 years up to the baseline visit. Diabetes mellitus was defined as a previous diagnosis of diabetes, fasting glucose ≥ 126 mg/dL, or use of glucose-lowering agents, including insulin and oral medication. Physical activity was measured using the Physical Activity Scale for the Elderly (PASE) [25]. Medications regularly used within the previous 30 days were recorded during clinic visits, and all prescribed and nonprescribed medications were stored in an electronic medication inventory database (San Francisco Coordinating Center, San Francisco, CA, United States).

### Statistical analysis

Participant characteristics were summarized using means and standard deviations (SDs), medians and interquartile ranges, or frequencies and percentages, as appropriate. This was an exploratory analysis, and no a priori primary exposure–outcome relationship was prespecified. Baseline characteristics were analyzed for the entire cohort and stratified according to HOMA-IR and TyG index quartiles. Differences between groups were assessed using analysis of variance for continuous variables and chi-squared tests for categorical variables. Cox proportional hazard models were used to estimate the risk of incident fractures, with hazard ratios (HR) and 95% confidence intervals (CI), with Q1 as the reference group. Model 1 was adjusted for age and clinical site. Model 2 was further adjusted for BMI. Model 3, the final multivariable model, was further adjusted for prevalent fractures, smoking status, history of cancer, rheumatoid arthritis, corticosteroid use, antiosteoporosis medication use, and total hip BMD as covariates. Assessment of the proportional hazards assumption indicated no violations of proportionality. Restricted cubic splines were used to examine whether the associations between HOMA-IR and TyG index with fracture risk were nonlinear. Knots were placed at the 25th, 50th, and 75th percentiles of HOMA-IR and the TyG index. The spline curve for the log HR of fracture vs HOMA-IR and TyG index was plotted. False discovery rate (FDR) correction was applied in post hoc analyses to account for multiple comparisons.

A sensitivity analysis was performed, excluding participants with diabetes. Although diabetes was not identified as a significant covariate in our univariate analysis, this approach was undertaken based on evidence from previous studies regarding the relationship between diabetes and fractures. To account for the potential competing effect of mortality, additional analyses were conducted using Fine and Gray subdistribution hazard models, treating death prior to fracture as a competing event. Statistical significance was set at *P* < .05, and all analyses were performed using SAS version 9.4 (SAS Institute Inc., Cary, NC, United States).

## Results

### Characteristics of participants

The characteristics of the participants according to the HOMA-IR and TyG index quartiles are summarized in Tables 1 and 2, respectively. In total, 5416 participants were included in the HOMA-IR analysis. Participants in the highest quartile (Q4) of HOMA-IR had the lowest mean age (72.6 years). BMI and triglyceride and glucose levels increased progressively across quartiles, with the highest values observed in Q4 (*P* < .001). The proportion of participants with diabetes was highest in Q4 (33.5%), while the PASE was lowest in Q4. BMD was the highest in Q4 across all measurement sites.

**Table 1:** Characteristics of participants grouped by HOMA-IR quartiles

Variables	Q1 (*N* = 1352)	Q2 (*N* = 1358)	Q3 (*N* = 1353)	Q4 (*N* = 1353)	*P*-value
Age, yr	74.2 ± 6.2	74.3 ± 6.1	73.3 ± 5.6	72.6 ± 5.3	<.0001
BMI, kg/m^2^	24.9 ± 2.8	26.5 ± 2.9	27.9 ± 3.3	30.3 ± 4.0	<.0001
Serum triglyceride, mg/dL	107.7 ± 55.7	133.2 ± 76.0	156.5 ± 86.1	204.2 ± 132.5	<.0001
Serum glucose, mg/dL	94.3 ± 11.3	100.3 ± 14.3	105.7 ± 19.0	122.1 ± 33.6	<.0001
Serum insulin, uIU/mL	4.02 ± 1.01	6.58 ± 1.04	9.48 ± 1.70	17.57 ± 8.09	<.0001
HOMA-IR	0.93 ± 0.23	1.60 ± 0.19	2.42 ± 0.31	5.24 ± 2.93	<.0001
Prevalent fracture	337 (25.0)	291 (21.5)	305 (22.6)	309 (22.9)	.320
Current smoking	53 (3.9)	46 (3.4)	50 (3.7)	38 (2.8)	.170
Alcohol consumption	964 (71.5)	906 (66.7)	869 (64.3)	783 (58.0)	<.0001
Physical Activity Scale	151.1 ± 70.8	147.5 ± 67.5	150.9 ± 64.9	138.5 ± 68.5	<.0001
History of any cancer	412 (30.5)	400 (29.5)	397 (29.3)	367 (27.1)	.070
Rheumatoid arthritis	68 (5.0)	56 (4.1)	63 (4.7)	96 (7.1)	.013
Diabetes mellitus	58 (4.3)	108 (8.0)	158 (11.7)	453 (33.5)	<.0001
Medication use					
Corticosteroid	108 (8.3)	123 (9.5)	107 (8.2)	113 (8.7)	.990
Thiazide diuretic	106 (8.1)	142 (11.0)	176 (13.5)	226 (17.3)	<.0001
Antiosteoporosis	36 (2.8)	27 (2.1)	19 (1.5)	23 (1.8)	.036
Bone mineral density, g/cm^2^					
L-spine	1.15 ± 0.26	1.17 ± 0.25	1.17 ± 0.23	1.20 ± 0.25	<.0001
Femoral neck	0.76 ± 0.12	0.77 ± 0.13	0.79 ± 0.13	0.82 ± 0.13	<.0001
Total hip	0.92 ± 0.13	0.94 ± 0.14	0.96 ± 0.14	1.00 ± 0.14	<.0001
Osteoporosis status					
Osteoporosis	97 (7.2)	80 (5.9)	53 (3.9)	45 (3.3)	<.0001
Osteopenia	622 (46.0)	567 (41.8)	558 (41.3)	451 (33.4)	
Normal	632 (46.8)	708 (52.3)	739 (54.7)	856 (63.3)	

Abbreviations: BMI, body mass index; HOMA-IR, Homeostatic Model Assessment of Insulin Resistance.

The data are presented mean ± SD or *N* (%).

HOMA-IR quartile range: Q1 (0.340-1.290), Q2 (1.291-1.936), Q3 (1.937-3.023), Q4 (3.024-33.723). Osteoporosis was defined according to WHO criteria (*T*-score≤ −2.5) at the femoral neck, calculated using a female-specific reference database derived from the NHANES white female population aged 20-29 years.

**Table 2:** Characteristics of participants grouped by TyG quartile

Variables	Q1 (N = 1347)	Q2 (N = 1345)	Q3 (N = 1346)	Q4 (N = 1346)	*P*-value
Age, years	74.5 ± 6.2	73.7 ± 5.9	73.4 ± 5.7	72.8 ± 5.5	<.0001
BMI, kg/m^2^	25.8 ± 3.3	27.0 ± 3.7	27.8 ± 3.7	29.0 ± 3.9	<.0001
Serum triglyceride, mg/dL	73.1 ± 14.9	108.9 ± 16.1	150.2 ± 26.2	269.8 ± 125.7	<.0001
Serum glucose, mg/dL	95.2 ± 9.5	99.6 ± 12.1	105.1 ± 17.1	122.4 ± 35.7	<.0001
Serum insulin, uIU/mL	6.38 ± 3.92	8.11 ± 4.85	9.92 ± 5.63	13.31 ± 8.80	<.0001
TyG index	4.41 ± 0.11	4.64 ± 0.05	4.82 ± 0.06	5.15 ± 0.20	<.0001
Prevalent fracture	302 (22.5)	343 (25.5)	313 (23.3)	272 (20.3)	.090
Current smoking	42 (3.1)	58 (4.3)	44 (3.3)	42 (3.1)	.640
Alcohol consumption	939 (69.8)	879 (65.5)	882 (65.5)	796 (59.3)	<.0001
Physical Activity Scale	148.6 ± 68.3	150.0 ± 68.8	147.4 ± 68.9	141.7 ± 66.3	.008
History of any cancer	410 (30.4)	384 (28.6)	414 (30.8)	361 (26.8)	.120
Rheumatoid arthritis	63 (4.7)	73 (5.4)	68 (5.1)	78 (5.8)	.270
Diabetes mellitus	52 (3.9)	94 (7.0)	181 (13.5)	448 (33.3)	<.0001
Medication use					
Corticosteroid	128 (9.9)	112 (8.6)	120 (9.3)	89 (6.9)	.016
Thiazide diuretic	119 (9.2)	140 (10.8)	158 (12.2)	230 (17.8)	<.0001
Antiosteoporosis	35 (2.8)	31 (2.4)	21 (1.7)	18 (1.4)	.008
Bone mineral density, g/cm^2^					
L-spine	1.15 ± 0.25	1.17 ± 0.25	1.17 ± 0.24	1.20 ± 0.25	.0001
Femoral neck	0.77 ± 0.12	0.78 ± 0.13	0.79 ± 0.13	0.81 ± 0.13	<.0001
Total hip	0.92 ± 0.13	0.94 ± 0.14	0.96 ± 0.14	1.00 ± 0.14	<.0001
Osteoporosis status					
Osteoporosis	102 (7.6)	66 (4.9)	56 (4.2)	47 (3.5)	<.0001
Osteopenia	571 (42.5)	580 (43.2)	559 (41.6)	477 (35.5)	
Normal	671 (49.9)	698 (51.9)	730 (54.3)	819 (61.0)	

Abbreviations: BMI, body mass index; TyG, triglyceride–glucose index.

The data are presented mean ± SD or *N* (%).

TyG quartile range: Q1 (3.7790-4.5480), Q2 (4.5481-4.7227), Q3 (4.7228-4.9331), Q4 (4.9332-6.5032). Osteoporosis was defined according to WHO criteria (*T*-score ≤ −2.5) at the femoral neck, calculated using a female-specific reference database derived from the NHANES white female population aged 20-29 years.

For the TyG index analysis, 5384 participants with available fasting triglyceride and glucose measurements were included, and their characteristics were consistent with those observed in the HOMA-IR analysis. Participants in Q4 of TyG index had the lowest mean age (72.8 years), while BMI, triglyceride and glucose levels, and the proportion of participants with diabetes were highest in Q4 (*P* < .001). Similarly, PASE was lowest in Q4, and BMD was highest across all measurement sites.

### Association of HOMA-IR with fracture risk

**Figure 1:**
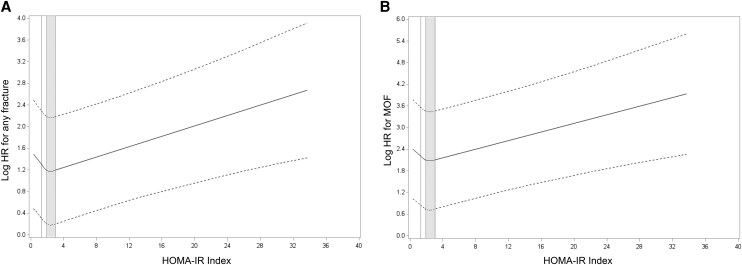
Spline plot of the log HR for (A) any fracture and (B) MOF by HOMA-IR index level. The model was adjusted for age, clinical site, body mass index, prevalent fractures, smoking status, history of cancer, rheumatoid arthritis, corticosteroid use, antiosteoporosis medication use, and total hip bone mineral density. The shaded area indicates the range that includes the third quartile (Q3).

During the follow-up period after baseline, 1418 men (26.2%) experienced at least one clinical fracture, including 341 men (6.3%) with at least one hip fracture, 243 men (4.5%) with at least one clinical vertebral fracture, and 754 men (13.9%) with at least one MOF. There was no evidence of an independent association of HOMA-IR quartile with risks of hip and clinical vertebral fractures, and limited evidence of associations of HOMA-IR quartile with risks of any fracture and MOF (Table 3). For any fracture, participants in Q3 compared with those in Q1 have a significantly lower risk across all models, including Model 1 (HR 0.79, 95% CI 0.68-0.92), Model 2 (HR 0.80, 95% CI 0.69-0.94), and Model 3 (HR 0.82, 95% CI 0.70-0.97). This association remained statistically significant after FDR correction. For clinical vertebral fractures, Q2 vs Q1 was associated with a significantly lower risk in Model 1 (HR 0.70, 95% CI 0.50-0.99); however, this association was no longer significant after further adjustments in Models 2 and 3. For MOF, Q3 compared with Q1 was associated with a significantly lower risk in Model 1 (HR 0.80, 95% CI 0.65-0.99), but this association was attenuated and became nonsignificant in Models 2 and 3. There was no evidence of an association between HOMA-IR and hip fracture. Using spline analyses, we identified a nonlinear, U-shaped relationship between HOMA-IR and fracture risk. The risks for any fracture (Fig. 1A) and MOF (Fig. 1B) were lowest at moderate HOMA-IR levels (range: 1.937-3.023, corresponding to Q3), with increased risks observed at both lower and higher HOMA-IR values. The equality-of-slopes assumption failed for HOMA-IR Q3 (1.937-3.023) vs Q4 (3.024-33.723) for any fracture and MOF (*P* = .01 for both outcomes), indicating that the association between HOMA-IR and the risk of any fracture and MOF differed above and below the cutoff point of 3.023.

**Table 3:** Association between HOMA-IR quartile and fracture incidence

Any fracture (*n* = 1418)			
HOMA-IR	Hazard ratio (95% CI)		
	Model 1	Model 2	Model 3
Q1	Ref.	Ref.	Ref.
Q2	0.88 (0.76, 1.01)	0.89 (0.77, 1.02)	0.92 (0.79, 1.07)
Q3	**0.79 (0.68, 0.92)**	**0.80 (0.69, 0.94)**	**0.82 (0.70, 0.97)**
Q4	0.88 (0.76, 1.02)	0.91 (0.77, 1.07)	0.93 (0.79, 1.11)

Hip fracture (*n* = 341)			
HOMA-IR	Hazard ratio (95% CI)		
	Model 1	Model 2	Model 3
Q1	Ref.	Ref.	Ref.
Q2	1.12 (0.85, 1.48)	1.16 (0.87, 1.54)	1.15 (0.85, 1.55)
Q3	0.73 (0.53, 1.00)	0.78 (0.56, 1.09)	0.77 (0.55, 1.09)
Q4	0.90 (0.66, 1.23)	1.02 (0.72, 1.45)	1.01 (0.70, 1.45)

Clinical vertebral fracture (*n* = 243)			
HOMA-IR	Hazard ratio (95% CI)		
	Model 1	Model 2	Model 3
Q1	Ref.	Ref.	Ref.
Q2	**0.70 (0.50, 0.99)**	0.73 (0.51, 1.03)	0.76 (0.53, 1.09)
Q3	0.77 (0.55, 1.09)	0.83 (0.58, 1.19)	0.92 (0.63, 1.33)
Q4	0.79 (0.55, 1.13)	0.90 (0.60, 1.36)	0.92 (0.60, 1.41)

Major osteoporotic fracture (*n* = 754)			
HOMA-IR	Hazard ratio (95% CI)		
	Model 1	Model 2	Model 3
Q1	Ref.	Ref.	Ref.
Q2	0.96 (0.79, 1.17)	0.98 (0.81, 1.19)	1.00 (0.81, 1.22)
Q3	**0.80 (0.65, 0.99)**	0.84 (0.67, 1.04)	0.86 (0.69, 1.07)
Q4	0.93 (0.76, 1.15)	1.00 (0.80, 1.27)	1.02 (0.81, 1.30)

Cox proportional hazards model was adjusted for age and clinical site (Model 1). Further adjustments were made as follows: Model 2: further adjusted for body mass index; Model 3: further adjusted for prevalent fractures, smoking status, history of cancer, rheumatoid arthritis, corticosteroid use, antiosteoporosis medication use, and total hip bone mineral density.

**Figure 2:**
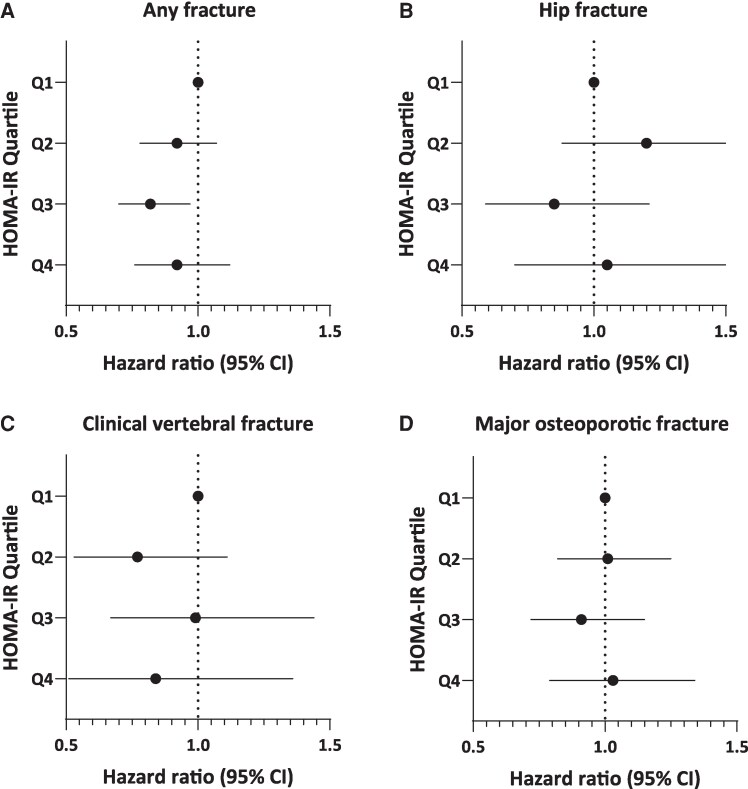
Association between HOMA-IR quartile and fracture incidence in sensitivity analysis excluding participants with diabetes. (A) Any fracture. (B) Hip fracture. (C) Clinical vertebral fracture. (D) Major osteoporotic fracture. Model 3, a multivariable-adjusted model, was used, adjusting for age, clinical site, body mass index, prevalent fractures, smoking status, history of cancer, rheumatoid arthritis, corticosteroid use, antiosteoporosis medication use, and total hip bone mineral density.

In the sensitivity analysis, after excluding participants with diabetes (Fig. 2), findings for any fracture were consistent with those of the primary analysis. In the fully adjusted Model 3 of the HOMA-IR analysis, participants in Q3 vs Q1 had a significantly lower HR for any fracture (HR 0.82, 95% CI 0.70-0.97) (Fig. 2). This finding was consistent with results from Model 3 of the primary analysis, which included participants with diabetes and similarly indicated a significantly lower HR for any fracture in Q3 vs Q1. As observed in Model 3 of the primary analysis for HOMA-IR, sensitivity analysis indicated no significant associations for hip fracture, clinical vertebral fracture, or MOF.

### Association of TyG index with fracture risk

**Figure 3:**
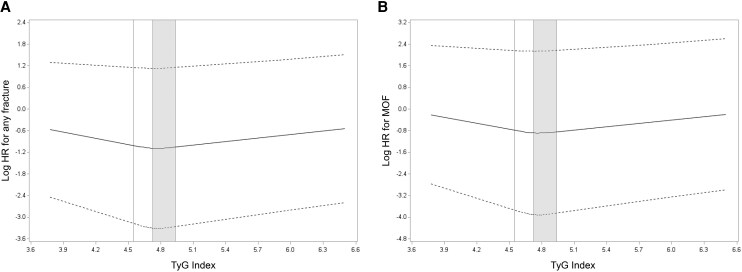
Spline plot of the log HR for (A) any fracture and (B) MOF by TyG index level. The model was adjusted for age, clinical site, body mass index, prevalent fractures, smoking status, history of cancer, rheumatoid arthritis, corticosteroid use, antiosteoporosis medication use, and total hip bone mineral density. The shaded area indicates the range that includes the third quartile (Q3).

Analysis of fracture incidence by TyG index quartiles revealed results similar to those observed in the HOMA-IR analysis (Table 4). During the follow-up period after baseline, 1406 men (26.1%) experienced at least one clinical fracture, including 336 men (6.2%) with at least one hip fracture, 240 men (4.5%) with at least one clinical vertebral fracture, and 746 men (13.9%) with at least one MOF. There was no evidence of associations of TyG quartile with risk of hip or clinical vertebral fracture. For any fracture, participants in Q3 vs Q1 had a significantly lower risk in all models, including Model 1 (HR 0.83, 95% CI 0.71-0.96), Model 2 (HR 0.83, 95% CI 0.72-0.97), and Model 3 (HR 0.84, 95% CI 0.72-0.98). This association remained statistically significant after FDR correction. For MOF, Q3 compared with Q1 was associated with a significantly lower risk in Model 1 (HR 0.81, 95% CI 0.67-0.99), and this association remained significant in the full multivariate model (Model 3) (HR 0.80, 95% CI 0.65-0.99). However, after FDR correction, the association between Q3 compared with Q1 and MOF was no longer significant (FDR-adjusted *P* = .076). There were no associations between TyG index and either clinical vertebral or hip fractures. Spline analyses indicated a nonlinear, U-shaped association between the TyG index and fracture risk (Fig. 3). The risk of any fracture (Fig. 3A) and MOF (Fig. 3B) was lowest at moderate TyG index levels (range: 4.7228-4.9331, corresponding to Q3), with higher risk observed at both lower and higher levels. The equality-of-slopes assumption held for TyG index and both any fracture and MOF outcomes.

**Table 4:** Association between TyG index quartile and fracture incidence

Any fracture (*n* = 1406)			
TyG	Hazard ratio (95% CI)		
	Model 1	Model 2	Model 3
Q1	Ref.	Ref.	Ref.
Q2	0.89 (0.77, 1.03)	0.89 (0.77, 1.03)	0.88 (0.76, 1.03)
Q3	**0.83 (0.71, 0.96)**	**0.83 (0.72, 0.97)**	**0.84 (0.72, 0.98)**
Q4	0.90 (0.78, 1.04)	0.91 (0.79, 1.07)	1.00 (0.85, 1.17)

Hip fracture (*n* = 336)			
TyG	Hazard ratio (95% CI)		
	Model 1	Model 2	Model 3
Q1	Ref.	Ref.	Ref.
Q2	1.03 (0.77, 1.38)	1.05 (0.78, 1.41)	1.00 (0.74, 1.36)
Q3	0.98 (0.72, 1.32)	1.02 (0.75, 1.38)	0.93 (0.67, 1.27)
Q4	0.93 (0.68, 1.26)	1.00 (0.72, 1.38)	1.04 (0.75, 1.45)

Clinical vertebral fracture (*n* = 240)			
TyG	Hazard ratio (95% CI)		
	Model 1	Model 2	Model 3
Q1	Ref.	Ref.	Ref.
Q2	0.71 (0.51, 1.00)	0.73 (0.52, 1.03)	0.79 (0.56, 1.13)
Q3	0.72 (0.51, 1.03)	0.75 (0.53, 1.07)	0.85 (0.59, 1.23)
Q4	0.74 (0.52, 1.05)	0.78 (0.54, 1.14)	0.92 (0.63, 1.36)

Major osteoporotic fracture (*n* = 746)			
TyG	Hazard ratio (95% CI)		
	Model 1	Model 2	Model 3
Q1	Ref.	Ref.	Ref.
Q2	0.83 (0.68, 1.00)	0.83 (0.68, 1.01)	0.83 (0.68, 1.02)
Q3	**0.81 (0.67, 0.99)**	0.83 (0.67, 1.01)	**0.80 (0.65, 0.99)**
Q4	0.83 (0.68, 1.02)	0.86 (0.69, 1.06)	0.93 (0.75, 1.16)

Cox proportional hazards model was adjusted for age and clinical site (Model 1). Further adjustments were made as follows: Model 2: further adjusted for body mass index; Model 3: further adjusted for prevalent fractures, smoking status, history of cancer, rheumatoid arthritis, corticosteroid use, antiosteoporosis medication use, and total hip bone mineral density.

**Figure 4:**
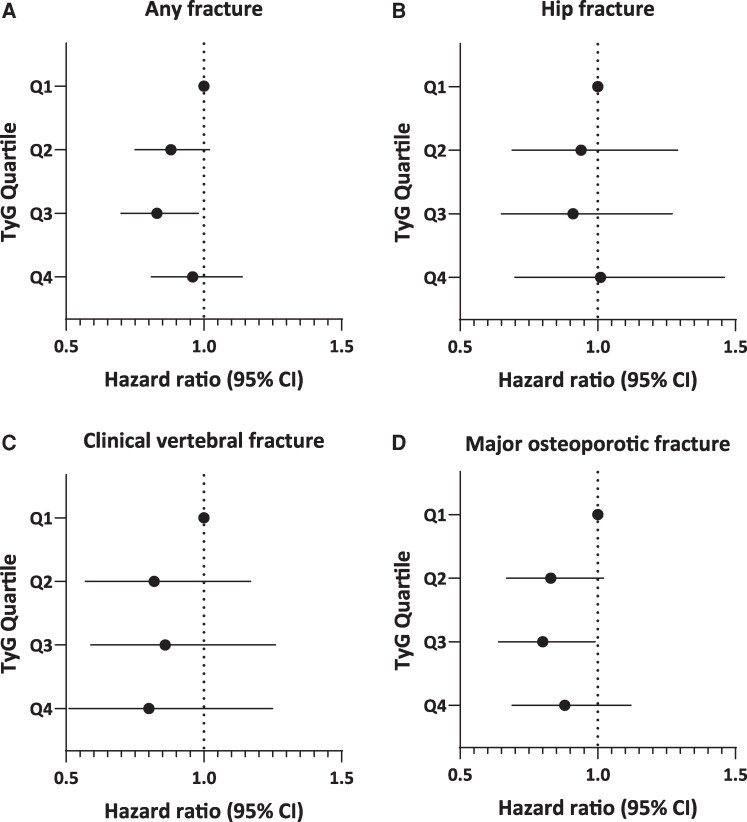
Association between TyG quartile and fracture incidence in sensitivity analysis excluding participants with diabetes. (A) Any fracture. (B) Hip fracture. (C) Clinical vertebral fracture. (D) Major osteoporotic fracture. Model 3, a multivariable-adjusted model, was used, adjusting for age, clinical site, body mass index, prevalent fractures, smoking status, history of cancer, rheumatoid arthritis, corticosteroid use, antiosteoporosis medication use, and total hip bone mineral density.

In the sensitivity analysis, after excluding participants with diabetes (Fig. 4), the findings regarding the associations of IR parameters with risks of fracture outcomes were consistent with those for the primary analyses. In the fully adjusted Model 3 of the TyG analysis, Q3 participants compared with those in Q1 had a significantly lower HR for any fracture (HR 0.83, 95% CI 0.70-0.98) and MOF (HR 0.80, 95% CI 0.64-0.99) (Fig. 4).

## Discussion

This study examined the association between IR, measured using HOMA-IR and TyG indices, and fracture risk in older community-dwelling men. Approximately 25% of the male cohort experienced at least one incident fracture during follow-up, representing a substantial fracture burden in this population. Men with moderate IR levels, particularly those in Q3, compared with those with lower IR levels (Q1), appeared to have a reduced risk for any fracture and MOF, but not for clinical vertebral or hip fracture. However, as the overall findings varied across quartiles, fracture types, and models, our results suggest a complex and nonlinear relationship between IR and skeletal health.

Previous studies examining the relationship between IR and bone health have reported mixed findings, with some studies showing inverse associations with BMD [26-28] and others reporting the opposite [10, 29, 30]. Although the underlying mechanisms have not been fully elucidated, physiological concentrations of insulin have been shown to enhance osteoblast proliferation, stimulate collagen synthesis, and inhibit osteoclast activity in vitro [31]. Additionally, both osteoblasts and osteoclasts express insulin receptors on their surfaces [32-34], and experimental evidence suggests that these receptors play critical roles in regulating cell proliferation, survival, and differentiation [8, 35]. However, the protective effects of insulin may not necessarily reduce fracture risk because of the adverse effects of chronic inflammation, advanced glycation end products, and lipid dysregulation associated with IR [11, 36].

Diabetes further complicates the relationship between IR and fracture risk. T2DM is associated with increased fracture risk in some [37-40], but not all studies [41, 42]. A study involving 2398 older adults without diabetes (53% women) found that higher HOMA-IR levels were associated with greater BMD, but were not consistently linked to reduced fracture risk after adjusting for BMI and BMD [10]. Additionally, some studies have suggested that fracture risk may decrease in individuals with prediabetes [43, 44] or metabolic syndrome [45], both of which are closely linked to IR. These findings underscore the complexity of the relationship between IR and skeletal health, highlighting the need for further studies examining this association across diverse metabolic states and populations.

Our study revealed a significant reduction in fracture risk in a specific quartile (Q3), which was consistently observed in analyses using both HOMA-IR and the TyG index. Reduced fracture risk was observed within an intermediate range of IR, without a clear dose–response gradient across quartiles. This pattern is consistent with a nonlinear association between IR and fracture risk. The HOMA-IR range in Q3 (1.937-3.023) likely reflects moderately increased IR, which may explain the reduced fracture risk observed in this group. Insulin enhances osteoblast activity, stimulates collagen synthesis, and inhibits osteoclast activity [31]. However, the absence of protective effects in the highest quartile (Q4) supports the hypothesis of a threshold effect, in which the systemic complications of severe IR outweigh its potential skeletal benefits. Although the study population overlapped substantially, HOMA IR and the TyG index reflect complementary dimensions of IR. HOMA IR primarily represents hepatic IR derived from fasting insulin and glucose concentrations, whereas the TyG index incorporates fasting triglycerides and reflects IR related to lipid metabolism. The concordant nonlinear patterns observed across the 2 indices provide a consistent interpretive context, and TyG may serve as a practical alternative when insulin measurements are unavailable.

This study has several strengths. We analyzed a large, well-characterized cohort with extended follow-up and rigorously adjudicated fracture outcomes. The use of validated indices, including HOMA-IR and the TyG index, enabled a robust assessment of IR. Notably, this study is the first to evaluate the association between the TyG index and fracture risk.

There are, however, several limitations to our study. Residual confounding due to unmeasured variables such as dietary intake and vitamin D levels may have influenced the results. Triglyceride, the key component of the TyG index, can be affected by factors including dietary intake, medication use, and physical activity. These influences may introduce variability and potentially distort the relationship between TyG index and specific outcomes. Similarly, HOMA-IR may be influenced by exogenous insulin use. However, our sensitivity analysis excluding individuals with diabetes yielded consistent results. In addition, the use of surrogate indices such as HOMA-IR and TyG index, rather than direct measures of IR, remains a potential limitation. The findings also have limited generalizability, as the cohort consisted exclusively of predominantly White older men. Fracture epidemiology and metabolic profiles may differ across geographic and population settings, and male-only data from diverse contexts remain limited. Race was not included as a covariate, and race-stratified analyses were not performed due to small numbers of non-White participants. Given established racial differences in fracture risk, unmeasured racial differences may have influenced the observed associations. Furthermore, the observational design restricted the ability to establish a causal relationship between IR and fracture risk. Since multiple statistical comparisons were conducted, some of the significant findings may have occurred by chance rather than representing a true underlying association. Post hoc analyses using FDR correction were performed to address multiple testing. After correction, the association of Q3 vs Q1 with any fracture remained statistically significant for both HOMA-IR and TyG index, whereas the association between Q3 of TyG index and MOF was attenuated and no longer significant. Despite these limitations, our findings suggest that moderate IR may be associated with reduced fracture risk in specific contexts; however, the observed associations are not sufficiently robust to inform clinical practice. In conclusion, this study found that older community-dwelling men in Q3 of the HOMA-IR and TyG indices had modest reductions in the risk of any fracture and MOF compared with those in Q1. However, these significant associations were not consistently observed across other quartiles or fracture types, underscoring the complex and potentially nonlinear relationship between IR and fracture risk. These findings should be interpreted cautiously and require validation in other populations to confirm their validity and better understand the underlying mechanisms.

## Data Availability

Original data generated and analyzed during this study are included in this published article or in the data repositories listed in References.
